# Evaluating the maintenance of disease-associated variation at the blood group-related gene *B4galnt2* in house mice

**DOI:** 10.1186/s12862-017-1035-7

**Published:** 2017-08-14

**Authors:** Marie Vallier, Maria Abou Chakra, Laura Hindersin, Miriam Linnenbrink, Arne Traulsen, John F. Baines

**Affiliations:** 1 Max Planck Institute for Evolutionary Biology, Evolutionary Genomics, Plön, Germany; 2 Institute for Experimental Medicine, Section of Evolutionary Medicine, Christian-Albrechts-University of Kiel, Kiel, Germany; 3 Max Planck Institute for Evolutionary Biology, Evolutionary Theory, Plön, Germany; 4 Donnelly Centre for Cellular and Biomolecular Research, University of Toronto, Toronto, Canada

**Keywords:** *B4galnt2*, Blood group, Host-pathogen interaction, Balancing selection, Trade-off, Evolutionary game theory, Wright-fisher process

## Abstract

**Background:**

*B4galnt2* is a blood group-related glycosyltransferase that displays cis-regulatory variation for its tissue-specific expression patterns in house mice. The wild type allele, found e.g. in the C57BL/6 J strain, directs intestinal expression of *B4galnt2*, which is the pattern observed among vertebrates, including humans. An alternative allele class found in the RIIIS/J strain and other mice instead drives expression in blood vessels, which leads to a phenotype similar to type 1 von Willebrand disease (VWD), a common human bleeding disorder. We previously showed that alternative *B4galnt2* alleles are subject to long-term balancing selection in mice and that variation in *B4galnt2* expression influences host-microbe interactions in the intestine. This suggests that the costs of prolonged bleeding in RIIIS/J allele-bearing mice might be outweighed by benefits associated with resistance against gastrointestinal pathogens. However, the conditions under which such trade-offs could lead to the long-term maintenance of disease-associated variation at *B4galnt2* are unclear.

**Results:**

To explore the persistence of *B4galnt2* alleles in wild populations of house mice, we combined *B4galnt2* haplotype frequency data together with a mathematical model based on an evolutionary game framework with a modified Wright-Fisher process. In particular, given the potential for a heterozygote advantage as a possible explanation for balancing selection, we focused on heterozygous mice, which express *B4galnt2* in both blood vessels and the gastrointestinal tract. We show that *B4galnt2* displays an interesting spatial allelic distribution in Western Europe, likely due to the recent action of natural selection. Moreover, we found that the genotype frequencies observed in nature can be produced by pathogen-driven selection when both heterozygotes and RIIIS/J homozygotes are protected against infection and the fitness cost of bleeding is roughly half that of infection.

**Conclusion:**

By comparing the results of our models to the patterns of polymorphism at *B4galnt2* in natural populations, we are able to recognize the long-term maintenance of the RIIIS/J allele through host-pathogen interactions as a viable hypothesis. Further, our models identify that a putative dominant-, yet unknown protective function of the RIIIS/J allele appears to be more likely than a protective loss of intestinal *B4galnt2* expression in RIIIS/J homozygotes.

**Electronic supplementary material:**

The online version of this article (doi:10.1186/s12862-017-1035-7) contains supplementary material, which is available to authorized users.

## Background

Von Willebrand disease (VWD) is a common human bleeding disorder characterized by a defect of coagulation caused either by low plasma levels of von Willebrand factor (VWF) or a dysfunctional VWF. In a mouse model of VWD – the laboratory strain RIIIS/J – the disease is caused by a cis-regulatory mutation at the *B4galnt2* gene, a blood group related glycosyltransferase [1, 2]. This mutation switches the usual expression pattern of *B4galnt2* in the gastrointestinal (GI) epithelium, as observed in the wild type strain C57BL/6 J and other vertebrates [3], to the vascular endothelium in the RIIIS/J strain. Vascular expression of *B4galnt2* leads to aberrant glycosylation of VWF, resulting in its accelerated clearance from circulation. Accordingly, RIIIS/J mice have up to twenty times lower plasma levels of VWF than C57BL/6 J mice [2].

Despite the expected fitness cost of prolonged bleeding times for wild animals, the RIIIS/J allele is found in high frequencies in various wild populations of house mice and their relatives [4, 5]. Furthermore these populations show signs of long term balancing selection maintaining both C57BL/6 J and RIIIS/J allele classes for at least 2.8 Ma. Further, in a previous survey of *Mus musculus domesticus* populations, a partial selective sweep revealed a recent increase in RIIIS/J allele frequency in a population from Southern France, while the allele was absent from a German population [4]. This suggests that selective force(s) operating on *B4galnt2* alleles in Western Europe may differ according to space and/or time.

Genome-wide scans for balancing selection in the human genome [6–8] identified a moderate number of genomic regions, but nearly all of them are involved in immunity *lato* sensu, supporting the hypothesis that *B4galnt2* could be involved in host-pathogen interactions, as shown for other blood-group related genes [9, 10]. Laboratory experiments show that the absence of *B4galnt2*-associated GalNac residues on the GI mucosa results in an altered resident microbiota [11], and that this modified GI microbiota confers lower susceptibility to a model of *Salmonella typhimurium* infection [12]. On the other hand, bacteria such as *Staphylococcus aureus* are known to use VWF to invade the host and escape the immune system [13, 14]. Although experimental evidence is lacking, *S. aureus*’s ability to utilize VWF could be compromised in RIIIS/J allele-bearing mice due to the low plasma levels of VWF, and hence lead to protection against this pathogen. Thus, potential benefits of the RIIIS/J allele could reside either in the *gain* of vascular expression and/or in the *loss* of GI expression in mice homozygous for the RIIIS/J allele, which would be associated with resistance against systemic and/or intestinal pathogens, respectively.

Under the above hypothesis, heterozygous mice are of particular interest, as they express *B4galnt2* in both blood vessels and the GI tract (i.e. the two allele classes influence on tissue-specific expression patterns is co-dominant) [4], potentially incurring both the cost of bleeding and pathogen susceptibility. It is known that heterozygous mice have the same bleeding phenotype as RIIIS/J homozygotes (i.e. the RIIIS/J allele’s effect on VWF is dominant) [2, 4], although we have little information concerning the susceptibility of heterozygous mice to pathogens in the wild. Indeed, heterozygous mice could have the same level of protection as the RIIIS/J homozygotes (e.g. in the case of *S. aureus* using VWF directly to infect), whereas on the other hand they could display similar susceptibility to gut pathogens as the C57BL/6 J homozygotes (e.g. when a gut pathogen utilizes *B4galnt2* specific GalNac residues in the mucosa). Finally, heterozygous mice could have an intermediate phenotype compared to both homozygotes in terms of resistance or susceptibility to pathogens.

In this study, we set out to determine the conditions under which the trade-off between prolonged bleeding times and pathogen susceptibility leads to the maintenance of the RIIIS/J allele, and in addition extended a previous geographic survey of *B4galnt2* allele frequencies [4] to characterize spatial selection across Western Europe in more detail. Accordingly, we modeled the interaction between host and pathogen using an evolutionary game with a Wright-Fisher process [15]. Since mice are diploid sexual organisms, we modified the Wright-Fisher “asexual” random process to include diploid reproduction. Pathogens were modeled as an environmental variable, being either present or absent, with the possibility for the environment to change regularly from one state to another. Alternatively, we also relax this assumption and model the pathogens such that their population depends on frequencies of susceptible hosts. Although simplified, the model provides a method to disentangle the effects of genotypic costs and environmental variability on the host population. Moreover, the environmental model resembles a “trench warfare” dynamic (i.e. advances and retreats of resistance allele frequency due to costs in the absence of a pathogen [16, 17], which we might expect in the context of balancing selection acting on resistance/susceptibility alleles as may be the case at *B4galnt2* [4]. To identify the model parameters that best explain the natural population dynamics, we compared the simulated populations to the observed wild populations. We found that the genotype frequencies observed in nature were best explained by a model where heterozygotes are protected against infection with a pathogen in a frequency-dependent manner, and the cost of bleeding being half that of infection.

## Results

### Wild mice

**Fig. 1:**
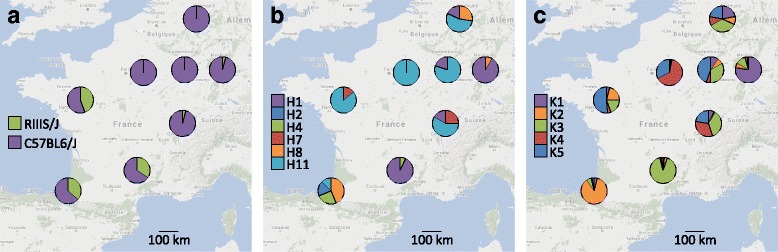
*B4galnt2* allele frequencies and population structure. **a**) *B4galnt2* allele frequency, **b**) Mitochondrial D-loop haplogroup, based on the haplogroup nomenclature from Bonhomme et al. [22], **c**) Distribution of genetic clusters based on STRUCTURE analysis applied to 18 neutral microsatellite markers, colors indicate genetic clusters. Map data: @2017 GeoBasis-DE/BKG (@2009), Google, Inst. Geogr. Nacional

First, in order to further characterize the intriguing geographic pattern of RIIIS/J allele frequency observed by Johnsen et al. [4], we typed *B4galnt2* allele classes using the same diagnostic PCR fragment in a set of eight wild population collections spread across France and Germany [18]. These populations represent six new locations, in addition to a resampling of the two locations previously analyzed by Johnsen et al. [4]. This reveals an intriguing pattern of distribution of the RIIIS/J allele: it is nearly absent in the north and east of France and in Germany, but it is consistently >30% in three local populations in the south and west of France (Fig. 1a).

**Fig. 2:**
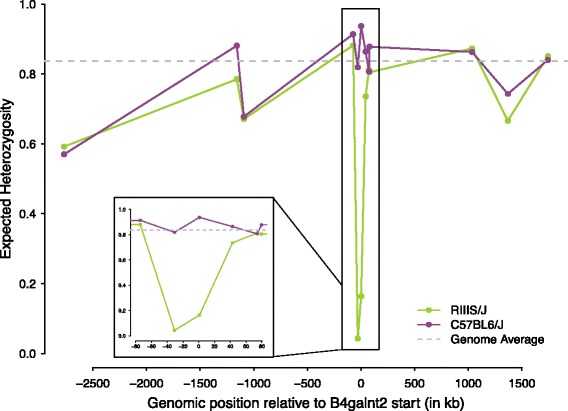
Expected heterozygosity in eight wild mice populations. Data are presented for 12 phased microsatellite loci spanning 160 kb surrounding the *B4galnt2* start position. The data are stratified for RIIIS/J (green) and C57BL/6 J (*purple*) allele classes. The genome average (*dotted gray lines*) is determined by 18 unlinked microsatellite loci

As we previously attributed differences in RIIIS/J allele frequency between two of these locations to a recent, partial selective sweep [4], we evaluated whether this pattern holds in this broader dataset. We thus typed 12 microsatellite loci linked to *B4galnt2* and resolved their haplotypic phase with respect to the RIIIS/J and C57BL/6 J alleles as previously described [4]. This reveals a near identical pattern (Fig. 2), whereby the expected heterozygosity of the two loci located closest to the cis-regulatory mutation of *B4galnt2* (−30 kb and 0 kb) is very low on the RIIIS/J background, while high and close to the genome average (as determined by 18 unlinked microsatellites) [18] on the C57BL/6 J background.

Due to the extremely high level of nucleotide divergence observed between the RIIIS/J and C57BL/6 J alleles, it is possible, however, that the two microsatellites displaying very low heterozygosity on the RIIIS/J background could have experienced e.g. one or more mutation interrupting their repeats and thus changing their mutation rate. Thus, we also performed direct Sanger sequencing of these individuals, which revealed no evidence of interruption. Rather, these two dinucleotide loci display a repeat number within the range of the alleles found on the C57BL/6 J background, but with very few alleles (two and four alleles at each locus, respectively). Thus, the pattern of a drastic, local reduction of microsatellite variability near the cis-regulatory mutation on the background of the RIIIS/J allele is most consistent with a partial selective sweep.

A second alternative is that the above-mentioned geographic pattern of allele frequency distribution could also be related to underlying population structure. Indeed, different waves of migration led to the colonization of Western Europe by house mice [19–21]: one coming from the east through modern day Turkey and Greece, and another from the south through North Africa and Spain. These migration routes led to the distinct maternal lineages present in Northern Europe and the Mediterranean basin [22, 23]. To test whether the distribution of *B4galnt2* alleles might be explained by population structure, we compared the observed allele frequencies to the previously established distribution of the mitochondrial D-loop haplogroups (Fig. 1b) and the genetic clusters identified by 18 nuclear microsatellite markers (Fig. 1c) [18]. This reveals little to no correspondence, e.g. some local populations dominated by the same mitochondrial haplogroup and/or genetic cluster display contrasting RIIIS/J frequencies, and on the other hand, local populations with similar RIIIS/J frequencies display contrasting haplogroups and/or genetic clusters. Thus, the observed pattern of RIIIS/J allele frequency appears to have little to do with underlying population structure.

Taken together, these results confirm and extend those of Johnsen et al. [4]: a partial selective sweep visible through the *B4galnt2*-linked microsatellites indicates that the RIIIS/J allele recently rose in frequency in Southwestern France, most likely due to the action of strong natural selection.

### Model

#### Constant environment

Although a constant environment is very unlikely in nature, the study of this limiting case allows us to test the behavior of our model. We chose to vary the costs of bleeding and infection from 0 to 1, incremented by steps of 0.2.

**Fig. 3:**
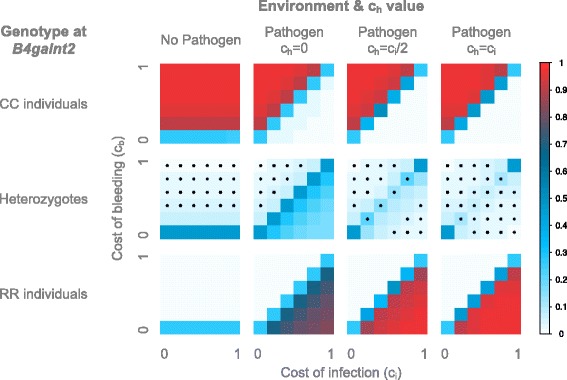
Average genotype frequencies in the model with a constant environment. The frequencies are displayed according to the cost of bleeding (y axis) and of infection (x axis). For the non-pathogenic environment, all c_h_ values are equivalent since c_i_ = c_h_ = 0 in all cases. For the pathogenic environment models with varying c_h_ values are shown. The average genotype frequencies across 50 simulations, each with 10,000 generations, are displayed. The frequencies are color-coded according to the legend on the right. Stars indicate an excess of homozygotes

In a constant environment with no pathogen (Fig. 3), the cost of infection c_i_ is irrelevant, and only the cost of bleeding c_b_ influences the outcome of the simulation. Thus, the three investigated values of the infection cost for the heterozygotes, c_h_ (equivalent to the dominance coefficient of a resistance phenotype in heterozygotes, see “Host” section in the Methods), lead to the same results, as c_h_ = c_i_ = 0. When c_b_ = 0, we have a neutral state (i.e. all individuals have the same fitness) leading to ~50% heterozygotes and ~25% of each homozygote. When c_b_ > 0, as expected the CC individuals make up the majority of the population, representing over 80% of the individuals, whereas the RR individuals are very close to 0 and the heterozygotes remain in low frequency (<20%). These proportions depend on the value of c_b_. Indeed, when c_b_ increases, the selection strength increases, particularly on the heterozygotes, leading to a deviation from Hardy-Weinberg equilibrium (HWE) and an excess of the favored homozygotes -- the CC individuals.

In a constant environment with a pathogen, the value of c_h_ has a great influence on the population frequencies, as it is dependent on the cost of infection. With c_h_ = 0 (Fig. 3), heterozygotes have the same fitness as the RR individuals, and the neutral state is reached whenever c_b_ = c_i_. Consistently, when c_b_ > c_i_, the CC individuals make up the majority of the population and when c_i_ > c_b_, the RR individuals and heterozygotes represent the majority. Interestingly, when the difference in costs becomes too high, the selection becomes so strong that the population deviates from HWE with an excess of homozygotes. However, this is only true when c_b_ > c_i_ but not when c_b_ < c_i_, indicating the asymmetry of the system that translates to a stronger effect of c_b_ compared to that of c_i_.

When c_h_ > 0 (Fig. 3) the heterozygotes have a lower fitness than both homozygotes, resulting in the population being mostly composed of the favored homozygotes (RR individuals when c_b_ < c_i_ and CC individuals when c_i_ < c_b_). Notably, the neutral state is reached only for the two extreme cases were all individuals have the same fitness (c_b_ = c_i_ = 0 and c_b_ = c_i_ = 1) and not for every c_b_ = c_i_ as in the previous model. This can be explained by the strong selection acting on the heterozygotes when c_h_ > 0, as they bear the dual cost of bleeding and infection. This leads to a deviation from HWE and an excess of homozygotes. As previously observed, this deviation is also present when the difference in costs becomes too strong, but this time for both c_i_ > c_b_ and c_i_ < c_b_. However, due to the asymmetry of the system when c_h_ = c_i_/2, the difference in costs must be stronger for c_i_ > c_b_ to lead to a deviation from HWE than for c_b_ > c_i_. For c_h_ = c_i_, the system becomes symmetrical: c_i_ and c_b_ have the same effect on the selection strength, leading to a deviation from HWE for the same difference in costs when c_i_ > c_b_ as when c_b_ > c_i_.

#### Changing environment

To approach the trench warfare dynamics that may be relevant for the putative host-pathogen interactions involving *B4galnt2*, we modeled the pathogen as an exogenous variable, being either present or absent from the environment. This property of the environment was regularly alternated according to host generations: the environment switches from pathogenic to non-pathogenic and back every S host generations. We investigated a broad range of switching frequencies: every 1, 10, 50, 100, 500, 1000 and 5000 host generations. For all values of c_h_, the two rapid switching frequencies (1 & 10) show similar results, as do the intermediate (50 & 100) and slow ones (500 onwards), thus, we display the results for 1, 50 and 500.

**Fig. 4:**
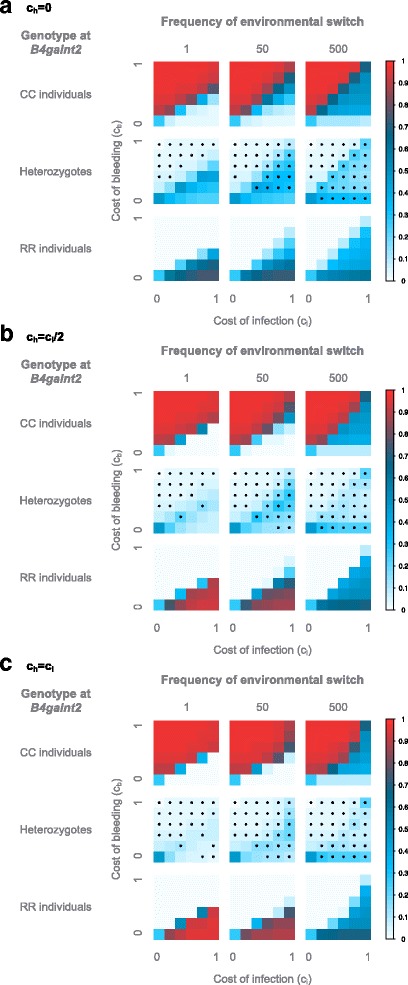
Average genotype frequencies in the model with a switching environment. The frequencies are displayed according to the frequency of environmental change expressed in host generations, the cost of bleeding (y axis) and of infection (x axis). The average genotype frequencies across 100 simulations, each with 10,000 generations, are displayed for c_h_ = 0 (**a**), c_h_ = c_i_/2 (**b**) and c_h_ = c_i_ **(C)**. The frequencies are color-coded according to legend on the right. Stars indicate an excess of homozygotes

First, we observe for c_h_ = 0 (Fig. 4a) that the parameter space is divided in two distinct regions where a given genotype is favored, as in the case for the constant pathogenic environment described previously. These two regions are separated by a boundary line where all genotypes coexist. In the constant pathogenic environment described previously, this boundary line represented the neutral state, where all genotypes have the same fitness, and the population is composed of ~50% heterozygotes and ~25% of each homozygotes. In the case of a changing environment however, this boundary line is different, as it does not represent a neutral state, but is still characterized by the coexistence of the three genotypes. Interestingly, the boundary lies around c_b_ = c_i_/2 for rapid switching, but approaches the c_b_ = c_i_ line for slower frequencies. Of note, this boundary region is important as it regularly occurs in the subsequent analyses and is in most cases characterized by the coexistence of all three genotypes, with only one- or a combination of two genotypes being favored above- or below the boundary region, respectively. In the case of rapid switching (Fig. 4a), the results are qualitatively very similar to the constant pathogenic environment: the boundary line approach a neutral state, since we have ~50% heterozygotes and ~25% of each homozygote; above this line, the population is composed of mostly CC individuals, and below this line, the RR individuals and heterozygotes represent the majority. The position of the line is however not the same. Indeed, the average payoff of the CC individuals in this model is c_i_/2, as half of the time these individuals bear no cost, and the other half they bear the cost of infection c_i_. This pushes the boundary line to c_b_ = c_i_/2 rather than c_b_ = c_i_ as in the constant pathogenic environment. We observe, like in the constant pathogenic environment, a deviation from HWE for c_b_> > c_i_/2. In this rapid model, the heterozygotes can be seen as an allelic pool that helps the system maintain both alleles in the population, and ensure the transition between the two homozygous states. For slower frequencies of environmental (pathogenic) change (Fig. 4a), the delay between switches is long enough for the alleles to fix, and for each period the system reaches the characteristics of the corresponding constant environment, therefore bringing the boundary line back towards c_b_ = c_i_, similar to the constant pathogenic environment. Moreover, under these conditions heterozygotes are no longer needed to maintain both alleles in the population, as the selection is strong enough to recover the alleles from very low frequencies. It appears that the heterozygotes even suffer from stronger selective pressure than the homozygotes, as we observe a deviation from HWE with an excess of homozygotes already with low fitness costs. Moreover, the asymmetry of the system is different compared to the constant environment. Indeed, in a non-changing environment, the influence of c_b_ compared to c_i_ on the selection strength is stronger, leading to deviations from HWE for smaller differences in costs when c_i_ < c_b_ than when c_b_ < c_i_. However, in this fluctuating environment a certain difference in costs is needed above the boundary line to lead to a deviation from HWE, as for the constant environment, but below the boundary it seems that only the value of c_b_ is important.

For c_h_ = c_i_/2 (Fig. 4b), the selection on the heterozygotes is stronger than on the homozygotes, as already observed under the constant environment. This leads to the “disappearance” of heterozygotes on the boundary line when c_b_ and c_i_ increase and an excess of homozygotes. The position of the line is however similar to that of the c_h_ = 0 model: it lies around c_b_ = c_i_/2 for the rapid environmental changes and approaches c_b_ = c_i_ for slowly fluctuating environments. For the rapidly switching environment (Fig. 4b), when c_b_ > c_i_/2 the CC individuals make up the majority of the population, as observed for c_h_ = 0. When c_b_ < c_i_/2 however, the population is composed of mostly RR individuals alone and not in conjunction with the heterozygotes as for c_h_ = 0. This is due to the lower fitness of the heterozygotes. A similar pattern is observed for the intermediate environment (Fig. 4b), but the selection appears to be stronger for below the boundary, as we observe an excess of homozygotes. For the slowly switching environment (Fig. 4b), we still observe a majority of CC individuals above the boundary, but below the line the RR individuals do not take over and rather coexist with the CC individuals. The heterozygotes, however, are still in very low frequency due to their low fitness, leading to an excess of homozygotes. This is again due to the asymmetry of the system: the cost of bleeding is always present but the cost of infection is present only half of the time, leading to a stronger selective pressure from the bleeding phenotype than from infection.

For c_h_ = c_i_ (Fig. 4c), the trend is similar to that of c_h_ = c_i_/2, but the selective pressure is stronger on the heterozygotes than with c_h_ = c_i_/2, leading to deviations from HWE with lower values of c_b_ and c_i_. Interestingly, we observe that the boundary line is no longer characterized by a linear c_b_ = c_i_/2 relationship, but rather takes an exponential distribution. This might be due to the non-additive dual cost of the heterozygous mice.

#### Similarity to natural populations

One important goal of constructing our model is to compare its results to the pattern of *B4galnt2* allele frequencies observed among wild populations of mouse species belonging to the genus *Mus*, in order to understand the selective forces maintaining disease-associated variation at this locus. Accordingly, we evaluated a broad collection of populations (summarized in Table 1) from the current- and two previous studies [4, 5]: Johnsen et al. [4] and the current study provide data from a total 10 *M. m. domesticus* populations from Europe, Africa and North America, whereas Linnenbrink et al. [5] added an ancestral *M. m. domesticus* population (Iran) and data from other house mouse subspecies and their relatives, including *M. M. musculus* (Kazakhstan), *M. M. castaneus* (India) and *M. spretus* (Spain).

**Table 1:** Description of the wild house mice populations used in this study

Population		Species	Location	Study	Sample Size				RIIIS/J Allele Frequency	Hardy-Weinberg
ID	Group				RR	RC	CC	Total		
DE	A	Mmd	Cologne-Bohn (DE)	i	0	0	36	36	0.00	Equilibrium
CB	A	Mmd	Cologne-Bohn (DE)	iii	0	0	15	15	0.00	Equilibrium
DB	A	Mmd	Divonne-lès-Bains (FR)	iii	0	1	11	12	0.04	Equilibrium
LO	A	Mmd	Louan-Villegruis-Fontaine (FR)	iii	0	0	12	12	0.00	Equilibrium
NA	A	Mmd	Nancy (FR)	iii	0	0	12	12	0.00	Equilibrium
SL	A	Mmd	Schömberg (DE)	iii	0	1	11	12	0.04	Equilibrium
Overall	A	--	--	--	0	2	97	99	0.01	Equilibrium
MC	B	Mmd	Massif Central (FR)	iii	1	11	7	19	0.34	Equilibrium
ES	B	Mmd	Espelette (FR)	iii	3	10	9	22	0.36	Equilibrium
AN	B	Mmd	Anger (FR)	iii	4	8	6	18	0.44	Equilibrium
CH	B	Mmd	Chicago (USA)	i	1	3	6	10	0.25	Equilibrium
CA	B	Mmd	Cameroon	i	2	18	9	29	0.38	Equilibrium
FR	B	Mmd	Massif Central (FR)	i	12	16	22	50	0.40	Homozygotes Excess
Overall	B	--	--	--	23	66	59	148	0.38	Equilibrium
IR	C	Mmd	Iran	ii	10	5	2	17	0.74	Equilibrium
SP	C	Ms	Spain	ii	19	7	1	27	0.83	Equilibrium
Overall	C	--	--	--	29	12	3	44	0.80	Equilibrium

Data from populations belonging to *Mus musculus* domesticus (Mmd) or *Mus spretus* (Ms) were included from (i) Johnsen et al. 2009 [4], (ii) Linnenbrink et al. 2011 [5] and (iii) Linnenbrink 2013 et al. [18]. The populations are categorized into three groups: A) populations with low RIIIS/J allele frequency, suspected to be in a non-pathogenic environment, B) populations with intermediate RIIIS/J allele frequency, suspected to be in a pathogenic environment and C) populations with high frequency of a modified RIIIS/J allele assumed to carry no bleeding cost, and suspected to be in a pathogenic environment. The sample size is given with the number of individuals of each genotype (RR for RIIIS/J homozygotes, CC for C57BL/6 J homozygotes and RC for heterozygotes), and the total number of mice. The corresponding RIIIS/J allele frequency and whether the population significantly deviates from HWE are also indicated

The study by Linnenbrink et al. [5] revealed that greater allelic and functional diversity is present at *B4galnt2* than that previously observed in derived *M. m. domesticus* populations. Indeed, the *M. m. domesticus* population from Iran and *M. spretus* population from Spain both display a modified RIIIS/J allele, which appears to turn off gastrointestinal expression of *B4galnt2* without turning on vascular expression. Interestingly, the frequency of this modified RIIIS/J allele class is higher than in any of the derived *M. m. domesticus* populations, which is consistent with it having the potential to be beneficial against infections without incurring the cost of prolonged bleeding times. Further, the *M. M. musculus* population from Kazakhstan contains yet another allele class at low frequency, termed “CRK”, which appears to be a recombinant allele driving expression in both the GI tract and blood vessels. For simplicity, however, we did not consider the Kazakh population containing this low frequency CRK class in the analysis. The *M. M. castaneus* population from India was also excluded, as no functional data on *B4galnt2* expression patterns is available for this population/subspecies. Thus, we ultimately grouped the included populations into three categories, summarized in Table 1:

A. Populations where the RIIIS/J allele is either absent or its frequency is very low, which are assumed to be in a non-pathogenic environment. These include five *M. m. domesticus* populations across Germany and Northeastern France, one of which (Cologne-Bonn) was sampled twice;
B. Populations displaying intermediate RIIIS/J allele frequencies, which are assumed to be in a pathogenic environment. These include one *M. m. domesticus* population from North America, one *M. m. domesticus* population from Africa, and three *M. m. domesticus* populations from Southwestern France, one of which (Massif Central) was sampled twice;
C. Populations with a modified RIIIS/J allele that are assumed to carry no bleeding cost and likewise assumed to be in a pathogenic environment. These include one *M. m. domesticus* population from Iran and one *M. spretus* population from Spain.

To evaluate which model and parameters best explain the observations in natural populations, we estimated the similarity between the simulated and observed genotype frequencies (see Methods).

**Fig. 5:**
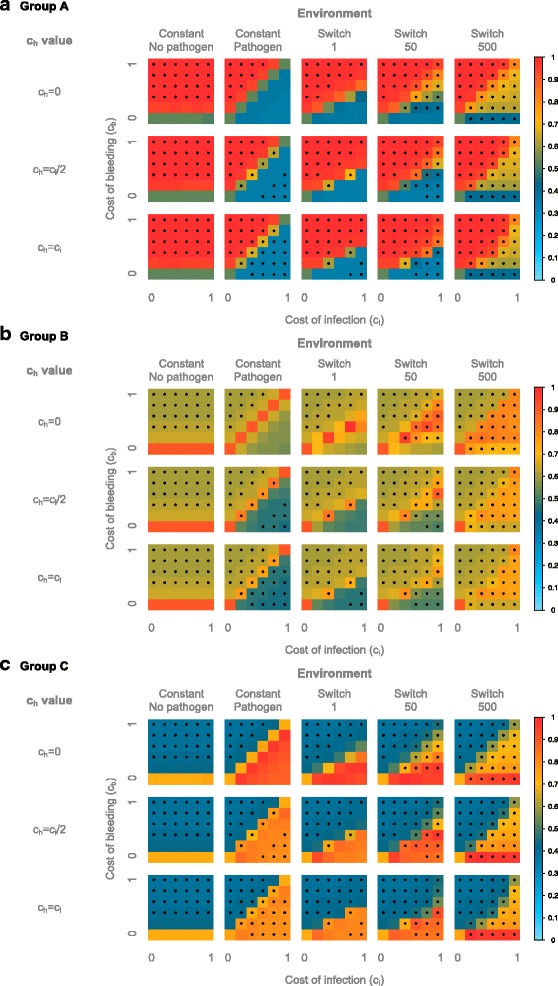
Similarity of the simulated populations to the natural populations. **a**) Similarity to populations from Group A, **b**) Similarity to populations from Group B, **c**) Similarity to populations from Group C. The similarity is displayed according to the value of c_h_, the cost of bleeding (y axis) and of infection (x axis), and the modeled environment (constant with- or without pathogen, and switching between a pathogenic- and non pathogenic environment every 1, 50 or 500 host generations). The similarity is color-coded according to the legend on the right. The similarity is calculated as one minus the average absolute difference between the simulated and natural genotype frequencies, hence a similarity of 1 is achieved when the genotype frequencies of the simulated populations are equal to that of the natural populations. Stars denote an excess of homozygotes

First, we observe for the populations assumed to be in a non-pathogenic environment (population group “A”) (Fig. 5a) that the simulations from the constant, non-pathogenic environment match well with the observed populations whenever c_b_ > 0 and for every value of c_h_. Notably, when c_b_ is very strong (>0.2) the population deviates from HWE. Further, the simulations from the constant pathogenic environment and the changing environments all match well with the observed data above the boundary line, for every value of c_h_. This might be explained by the asymmetry of the model, which generally favors CC individuals, yielding all simulations above the boundary to closely match the observed group A populations. Notably, the majority of the simulated population deviate from HWE, since only a small cost window above the boundary line is in equilibrium for the constant pathogenic environment and the rapidly switching one, when c_h_ < c_i_. For the slowly switching environments, only small values of c_b_ leave the population in equilibrium.

For the populations assumed to be in a pathogenic environment (population group “B”) (Fig. 5b), we observe that the constant non-pathogenic environment does not explain the observed data very well, only in the case where c_b_ = 0, which is likely unrealistic. For the constant pathogenic- and the switching environment, we observe that in general the best match to the real populations is at the boundary line, with the populations simulated in a rapidly changing environment for c_h_ = 0, reaching the highest similarity to the observed populations. Notably, only the constant pathogenic- and rapidly switching environments with c_h_ = 0 maintains HWE while providing relatively high similarity to the observed populations.

Finally, for the populations without bleeding phenotype (population group “C”) (Fig. 5c), we observe that the constant non-pathogenic environment is unlikely to explain the observed data: for c_b_ > 0 the similarity is between 40 and 50%, although it reaches 70% for c_b_ = 0. The constant pathogenic- and switching environment best explain the data below the boundary line, and it seems, as for the pathogenic populations, that these environments, are more likely to fit the real populations with c_h_ = 0 than with c_h_ > 0, as their genotype frequencies are very close to the observed ones (>90% similarity). The intermediate environment (S = 50) with c_h_ = 0 and the slowly switching environment (S = 500) with all c_h_ values both fit the populations well for null or very low values of c_b_, which is also consistent with our hypothesis that these mice carry no cost of bleeding. Notably, only the slowly switching environment produces high similarity with an excess of homozygotes, whereas the other environments produce high similarity while maintaining HWE.

Since all studied populations bear similar *B4galnt2* alleles, with the exception of the modified RIIIS/J allele found in group C, it is reasonable to assume that the populations will bear largely the same fitness costs. This applies to the C57BL/6 J allele class in all three groups regarding the cost of infection, c_i_. Similarly, we can consider population groups A and B to carry the same cost of bleeding, c_b_ > 0, whereas this is expected to be zero for group C. These assumptions allow us to further identify combinations of costs of bleeding and infection that might take place in nature, by comparing the similarity values across the three groups. First, group B can be seen as the “limiting factor” since they are approached by the simulated populations only at the boundary line in a rapidly changing environment and with c_h_ = 0. This reduces the space of possible cost parameters to c_b_ = c_i_/2, excluding the special case of c_b_ = c_i_ = 0. Population groups A and C are however never approached by the simulated populations at the boundary, but always above or below this line, respectively. This suggests that group A is in a constant environment without a relevant pathogen. There are however multiple possibilities for group C: assuming that c_b_ is indeed zero in these populations, a constant environment, a rapidly changing environment, and an intermediate frequency environment are all capable of producing the observed genotype frequencies. This is limited to small non-zero values of c_i_ in the constant environment, but is true for all c_i_ > 0 for the switching environments.

#### Pathogen as frequency

The models we investigated so far are important to understand the behavior of the system, approach trench warfare dynamics and model seasonal changes, but another key biological aspect is the reaction of a pathogen to the changes in host genotype frequencies. Thus, to address this aspect we modified our model to let the pathogen population vary according to the host population. For this, we express the pathogen as the proportion of susceptible individuals in the host population. Interestingly, this model does not lead to a trench-warfare dynamic, but quickly reaches an equilibrium that is maintained over 10,000 host generations.

**Fig. 6:**
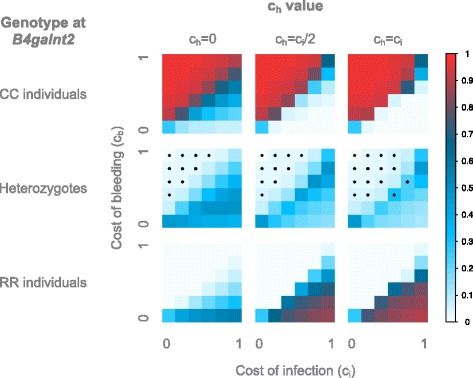
Average genotype frequencies in the model with a frequency-dependent environment. The frequencies are displayed according to the value of c_h_, the cost of bleeding (y axis) and of infection (x axis). The average frequencies across 100 simulations, each with 10,000 generations, are displayed. The frequencies are color-coded according to the legend on the right. Stars indicate an excess of homozygotes

First, we observe that the average population (Fig. 6) is relatively similar to the fast and intermediate environment (S = 1; S = 50). The selection strength appears however weaker, as more populations remain in HWE compared to the switching environments. For c_h_ = 0, the boundary line lies around c_b_ = c_i_/2, as for the rapidly switching environment. Above this line the population is composed of mostly CC individuals, whereas below the line RR individuals and heterozygotes share the majority. As for the rapidly switching environment, when c_b_ becomes too high compared to c_i_, the population deviates from HWE with an excess of homozygotes. For c_h_ = c_i_/2 however, the boundary appears to move towards c_b_ = c_i_. CC individuals represent the majority above this line, whereas RR individuals predominate below the line, as the selective pressures on the heterozygotes are stronger when c_h_ > 0. In contrast to the switching environment, where the boundary shows a deviation from HWE, we observe deviations from HWE in this model only above this line, suggesting that the selective pressures might be weaker in this model. For c_h_ = c_i_, the results are very similar to c_h_ = c_i_/2. However, with the selection strength being stronger on the heterozygotes, we see more deviations from HWE and a higher proportion of homozygotes above and below the boundary, as we previously observed for switching environments.

**Fig. 7:**
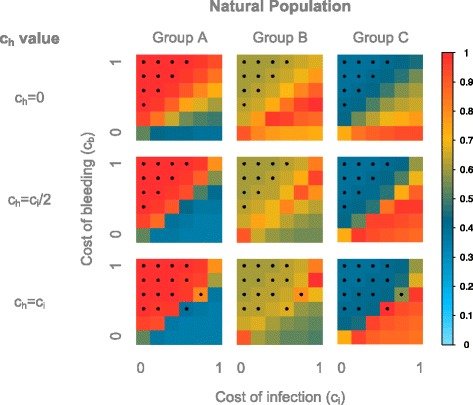
Similarity of the natural populations to the populations simulated in the model with frequency-dependent environment. The similarity is displayed according to the value of c_h_, the cost of bleeding (y axis) and of infection (x axis). The similarity is color-coded according to the legend on the right. The similarity is calculated as one minus the average absolute difference between the simulated and natural genotype frequencies, hence a similarity of 1 is achieved when the genotype frequencies of the simulated populations are equal to that of the natural populations. Stars indicate an excess of homozygotes

Given the resemblance of the frequency-dependent model to the switching environments in terms of genotype frequencies, we might expect similar results concerning the fit to the real populations. Indeed, we observe (Fig. 7) that the populations assumed to be in a non-pathogenic environment, group A, are best approximated when the costs are above the boundary line, regardless of the value of c_h_. For the populations assumed to be in a pathogenic environment, group B, the model fits best around the boundary in general, and in particular for c_h_ = 0. For the populations with no bleeding phenotype, group C, the models fit best below the boundary: for the special case of c_b_ = 0 with c_h_ = 0, and in a narrow space just below it with c_h_ > 0. As for the switching environment, comparing the results across populations enables us to infer the best overall model. Again, groups B and C are only compatible with c_h_ = 0. Group C is best approximated by the model for any c_i_ > 0.2, whereas group B is best approximated for c_b_ = c_i_/2 with c_i_ > 0.2. Group A is however not compatible with the two other population categories, as they are approximated by any c_b_ > c_i_/2 for c_h_ = 0, suggesting once again that they are associated with a non-pathogenic environment.

In conclusion, although conceptually very different from the exogenously changing environment, the frequency-dependent model leads to similar results as the rapidly changing environment. In both models it appears that the model with c_h_ = 0 is more plausible, with costs within the non-zero range of c_b_ = c_i_/2.

#### Hardy-Weinberg based process

Our modified Wright-Fisher based process represents a very good approximation of the real populations when considering genotype frequencies. In addition, we developed an alternative process based on HWE that calculates the expected number of individuals from each genotype based on the weighted fitness, which also represents a computationally faster model, as there is only one calculation and few random steps per generation.

Interestingly, this model leads to remarkably similar results to those obtained from the Wright-Fisher based process. The genotype frequencies are indeed very similar for both population dynamics model (constant environment: Additional file 1: Figure S1; switching environments: Additional file 2: Figure S2, frequency-dependent environment: Additional file 3: Figure S3) and consequently, the comparison to the natural populations is also very similar to the Wright-Fisher based process (constant & switching environment: Additional file 4: Figure S4, frequency-dependent environment: Additional file 5: Figure S5).

This model however represents a stronger selection regime, as the number of random steps is highly reduced compared to the Wright-Fisher based process. This translates into heterozygote frequencies being lower in the HWE-based process than in the random process for otherwise equal model parameters (c_b_, c_i_, c_h_, S), which consequently leads to an excess of homozygotes.

#### Effect of mutations/migration

The results presented thus far were produced using a mutation rate μ = 0.005, which in our system can also be viewed as a proxy for migration, as the two *B4galnt2* alleles considered are highly divergent including numerous SNPs and indels, which renders direct mutation from one functional allele class to the other unlikely. Since this mutation/migration rate could significantly impact the results, we investigated its effects using the HWE-based process.

**Fig. 8:**
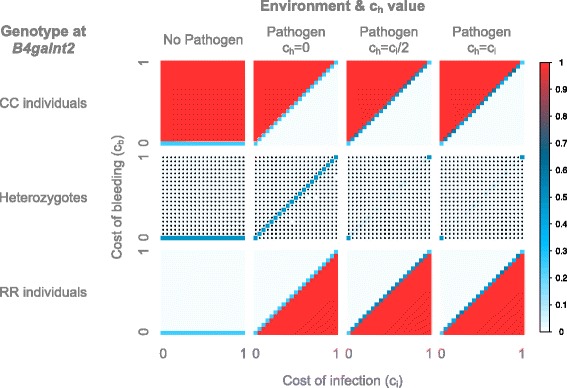
Average genotype frequencies in the model with a constant environment, without mutation. The frequencies are displayed according to the cost of bleeding (y axis) and of infection (x axis). For the non-pathogenic environment all c_h_ values are equivalent since c_i_ = c_h_ = 0 in all cases. For the pathogenic environment, models with varying c_h_ values are shown. The average genotype frequencies across 100 simulations using the HWE-process without mutation, each with 10,000 generations, are displayed. The frequencies are color-coded according to the legend on the right. Stars indicate an excess of homozygotes

For the constant environments (Fig. 8), we observe a relatively similar pattern as with mutation. In a constant non-pathogenic environment, we observe a neutral state for c_b_ = 0, and CC individuals make up the majority of the population when c_b_ > 0. For constant pathogenic environments, CC individuals predominate when c_b_ > c_i_ for every value of c_h_, as already observed in the model with mutations. When c_b_ < c_i_ and for every value of c_h_, RR individuals predominate, in contrast to the model with mutation, which leads to both the RR individuals and heterozygotes taking over the population for c_h_ = 0. In general, the model without mutation represents a very strong selection regime, as heterozygotes are nearly absent from every model, except for the neutral states, where all genotypes have equal fitness (c_b_ = 0 for constant non-pathogenic environment, c_b_ = c_i_ for constant pathogenic environment when c_h_ = 0, c_b_ = c_i_ = 0 and c_b_ = c_i_ = 1 for constant pathogenic environment when c_h_ > 0).

Although the starting environment has little influence on the long-term dynamics in the models with mutation, it has potentially strong consequences in the models without mutation. Therefore, it is necessary to distinguish the models that started in a pathogenic environment from those that started in a non-pathogenic environment for the exogenously changing environments.

**Fig. 9:**
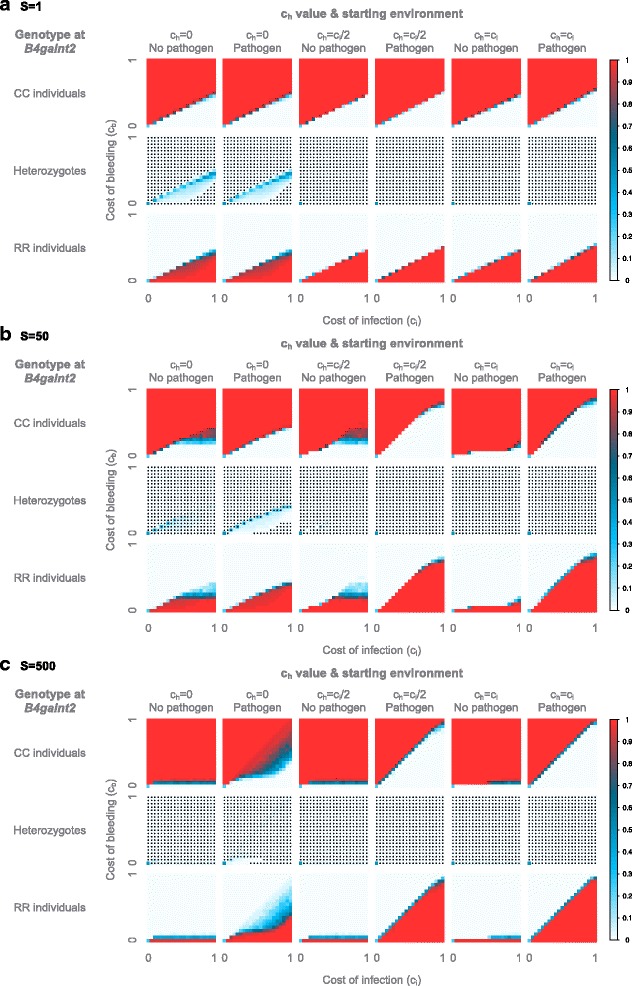
Average genotype frequencies in the model with a switching environment, without mutation. The frequencies are displayed according to the value of c_h_, the starting environment, the cost of bleeding (y axis) and the cost of infection (x axis). The environment switches every **a**) 1 host-generation **b**) 50 host-generations or **c**) 500 host-generations. The average genotype frequencies across 100 simulations using the HWE-process without mutation, each with 10,000 generations, are displayed. The frequencies are color-coded according to the legend on the right. Stars indicate an excess of homozygotes

For the rapidly switching environment (Fig. 9a), the starting environment has little influence on the results. These are however different from the models with mutation. Although the boundary line is at the same position, the heterozygotes are mostly in much lower frequency: they are limited to a narrow region around c_b_ = c_i_/2 for c_h_ = 0, and nearly absent for c_h_ > 0 (except for the neutral state c_b_ = c_i_ = 0), leaving the population to be comprised of mostly homozygotes.

For the intermediate environment (Fig. 9b), the starting environment has limited influence on the model with c_h_ = 0, but a strong influence on the models with c_h_ > 0. For the c_h_ = 0, the boundary line is at c_b_ = c_i_/2, as for the model with mutation, but like for the constant and rapid environment, the heterozygotes are very low in frequency except on the boundary. For c_h_ = c_i_/2, the simulations beginning in a non-pathogenic environment are relatively similar to those with mutation, but again the heterozygotes are nearly absent. For the simulations beginning with a pathogenic environment, the results are very similar to those from the constant pathogenic environment. Finally, for c_h_ = c_i_, both starting environments look very similar to the corresponding constant environment.

For the slowly switching environment (Fig. 9c), the starting environment strongly influences all models. For all values of c_h_, the models beginning in a non-pathogenic environment resemble the constant non-pathogenic environment, with the CC individuals representing the majority of the population for nearly all c_b_ > 0. For the simulations beginning in the pathogenic environment however, only models with c_h_ > 0 resemble the constant pathogenic environment. The model with c_h_ = 0 on the other hand differs, as the RR individuals make up the majority of the population only when c_b_ < c_i_/2, while both homozygotes are present when c_i_/2 < c_b_ < c_i_. This can be explained by the presence of heterozygote individuals. When the population begins in a pathogenic environment for c_h_ = 0, both RR individuals and heterozygotes are favored over CC individuals, leading to an initial phase where both RR individuals and heterozygotes are in high frequency in the population. The presence of heterozygotes allows the reappearance of CC individuals when the environment switches, and they subsequently become favored over both other genotypes. When c_h_ > 0 heterozygotes disappear quickly from the population as they have a lower fitness than both homozygotes. Thus, when the environment changes, the population is composed of only one homozygote genotype, and due to the absence of mutation the other genotypes are unable to return, hence the resemblance to the constant environment.

**Fig. 10:**
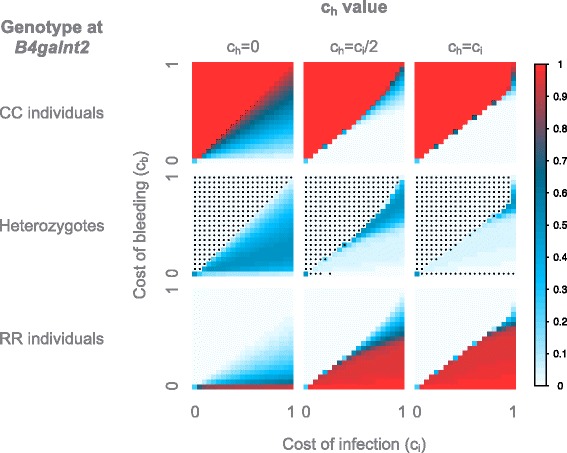
Average genotype frequencies in the model with a frequency-dependent environment, without mutation. The frequencies are displayed according to the value of c_h_, the cost of bleeding (y axis) and of infection (x axis). The average genotype frequencies across 100 simulations using the HWE-process without mutation, each with 10,000 generations, are displayed. The frequencies are color-coded according to the legend on the right. Stars indicate an excess of homozygotes

In contrast to the switching environments, the frequency-dependent environment without mutation (Fig. 10) is quite similar to the one with mutation. However, more populations deviate from HWE, and heterozygotes are in lower frequencies compared to the model with mutation.

In conclusion, mutation/migration appear to be dispensable to the maintenance of all three genotypes in the population only in the rapid and intermediate environments, both with c_h_ = 0 or in a frequency-dependent environment. Moreover, the starting environment, which has a negligible effect on the population frequencies when mutation is allowed, seems to dictate the genotype frequencies for intermediate and slowly switching environments in the absence of mutation.

## Discussion

The study of polymorphism at *B4galnt2* in house mice provides an interesting opportunity to elucidate the selective forces leading to the maintenance of disease-associated variation in nature. Previous studies revealed the action of long-term balancing selection on the one hand [5], in addition to dynamics on more recent timescales in the present study. Systematic review of signatures of balancing selection in the human genome identified genes involved in immunity *lato* sensu [6–8], indicating that host-pathogen interactions could be among the forces maintaining allelic diversity at *B4galnt2*. In this study, we set out to better understand the nature of potential trade-offs between resistance against pathogens and susceptibility to prolonged bleeding times surrounding variation at *B4galnt2*.

First, by extending our previous geographic survey of *B4galnt2* alleles across Western Europe from two- [4] to eight locations [18], we discovered an intriguing pattern of *B4galnt2* allele distribution, with northern populations being nearly devoid of the RIIIS/J allele in contrast to southwestern populations, which display intermediate RIIIS/J allele frequencies. Comparing this distribution of *B4galnt2* allele frequencies to population structure based on either mtDNA haplotypes or unlinked nuclear microsatellite markers [18] reveals little- to no correspondence. In contrast, we confirmed the pattern of a partial selective sweep of the RIIIS/J allele [4] in two additional populations, indicating that the high frequency of the RIIIS/J allele in the Southwestern populations is most consistent with the action of recent natural selection.

To further investigate host-pathogen interactions as a possible driver of selection at *B4galnt2*, we developed a mathematical model derived from the classical Wright-Fisher process, to which we added random mating. This was necessary as existing mathematical models, including the Wright-Fisher process are applicable to haploid hosts. Moreover, heterozygous individuals are of particular interest in this study system, as they express *B4galnt2* both in the blood vessels and the gastrointestinal tract, leaving them with a potential dual cost of prolonged bleeding and infection susceptibility. As our model focuses on the mechanisms maintaining host genotypes in a population, we treated the pathogen as an environmental probability p that can be constant (*p* = 1 - all susceptible hosts are infected; *p* = 0 - no host is infected), fluctuating (switching between 0 and 1 with frequency S expressed in host generations) or dependent on the proportion of susceptible individuals in the host population. We ran a very broad set of simulations, aiming to maximize the possible parameter combinations to fully understand the model and its behavior, and ultimately determine which parameters can lead to population frequencies similar to those observed in the wild.

Importantly, for nearly all combinations of parameters studied, we observe a similar dichotomy between regions of parameter space where a given genotype(s) is favored. The boundary between these regions can be observed at c_b_ = c_i_/2 or c_b_ = c_i_ (depending on the values of c_h_ and S), and is characterized by the coexistence of all three genotypes. The model is however asymmetric, as above the boundary, CC individuals always make up the majority of the population, while below the line RR individuals predominate, either alone or in conjunction with heterozygotes (depending on the values of c_h_ and S).

Interestingly, the two different population dynamic models used, a Wright-Fisher process with random mating or a HWE-based process, lead to remarkably similar results. The average genotype frequencies are similar in both models, with the differences being characterized by the variation around the mean being much larger in the random process, which contains 3 N random steps per generation, while the HWE-based process contains only a few. This result is quite valuable as it allows the use of the HWE-based model to quickly screen the parameter space to identify interesting parameter combinations, which can subsequently be run under the random process, which is much slower to compute due to its speed being proportional to population size.

Not surprisingly, the mutation rate can have a strong influence on the model, although the extent of this influence greatly depends on the environmental model considered. First, for constant-, frequency-dependent- or rapidly switching environments, the effect of mutation is rather weak. Indeed, for constant environments the direction of selection does not change over time, leading to the near fixation of a favored genotype, as the small number of unfavoured individuals produced through mutation is quickly removed by selection. In contrast, for rapidly switching environments the favored individuals do not have the time to take over the population before the environment changes, leading to the maintenance of all three genotypes. In this case, the switch from one homozygote to the other when the environment changes is ensured partly through the mating of heterozygotes and partly through mutation, which explains the limited influence of the latter on the population frequencies. For frequency-dependent environments, the system quickly reaches an equilibrium, which stems from the fitness of the different genotypes with little input from the mutation rate. Second, for slowly fluctuating environments the absence of mutation drastically influences the population. Indeed, when the environment changes infrequently, the favored genotype has sufficient time to take over, such that when the environment changes, only mutation can rescue the other genotypes in the population, leading to completely different population dynamics with- or without mutation. With mutation the system is reversible, and the genotypes can alternate according to the environment. Without mutation the population becomes rapidly fixed for one homozygous genotype and can no longer change, even when the environment switches between pathogenic and non-pathogenic states.

In the context of long-term balancing selection at *B4galnt2*, we use the mutation rate as a proxy for migration, since the high divergence between *B4galnt2* haplotypes and likely complex nature of the regulatory sequences separating them [24] make it unlikely that one allele would directly mutate to another. Moreover, *M. m. domesticus* populations in Western Europe display very little population structure on a continental scale [25], thus, models with migration are clearly more realistic.

Lastly, we compared the results of our models to the current and previous surveys [4, 5, 18] of DNA sequence polymorphism at *B4galnt2,* which we grouped in three categories: populations with very low RIIIS/J allele frequency, which are suspected to be in a non-pathogenic environment (group A); populations with intermediate RIIIS/J allele frequencies, which are suspected to be in a pathogenic environment (group B); and finally populations with high frequencies of a modified RIIIS/J allele that does not carry a cost of bleeding, which are suspected to be in a pathogenic environment (group C). For group B, the best fitting models are those with a rapidly switching environment and the frequency-dependent environment, each with costs of bleeding and infection on the boundary line (c_b_ = c_i_/2), excluding the special case of c_b_ = c_i_ = 0. For group C, the best fitting models are those with rapid- and intermediate switching environments and the frequency-dependent environment, for costs below the boundary (c_b_ < c_i_/2) and particularly for c_b_ = 0, which corresponds to prior knowledge of the expression phenotype for the modified RIIIS/J allele. For group A, any environment is suitable to explain the observed data, for any costs above the boundary.

With the exception of group C harboring a modified RIIIS/J allele class, the remaining populations studied share related alleles and their corresponding expression patterns, suggesting that they may have similar costs of bleeding and infection. For the three population groups to be compatible, the costs must follow a c_b_ = c_i_/2 relationship, excluding the c_b_ = c_i_ = 0 special case; group A must be in a constant non-pathogenic environment; groups B & C must be in a rapidly changing environment or frequency-dependent environment; and finally group C must have c_b_ = 0. It is notable that the model predictions perfectly match our hypotheses for groups A and C. Moreover, the predictions for group B (a frequency-dependent or rapidly switching environment) represent two biologically relevant models, the first one taking the response of the pathogen into account, while the second may reflect seasonal changes.

Interestingly, the better fitting model is that in which heterozygotes and RIIIS/J homozygotes are protected against bacterial infections (c_h_ = 0). Although our previous analysis of both commensal gut bacterial communities [11] and an experimental model of infectious gastroenteritis (*S. typhimurium*; [12]) suggest a potential benefit of the removal of *B4galnt2* expression in the GI tract, we note that *S. typhimurium* is not a naturally occurring mouse gut pathogen and requires antibiotic pre-treatment in order to cause intestinal pathology. On the other hand, although it played a comparatively smaller role, blood vessel expression driven by the RIIIS/J allele does appear to provide a small degree of protection in the *S. typhimurium* model, which might be associated with increased mucus thickness [12]. This indicates that the potential benefit of vascular *B4galnt2* expression does not reside solely in the blood vessels -- as could be the case with e.g. systemic infection with *Staphylococcus* -- but also in the gastrointestinal tract, where it seems to have a protective effect against *S. typhimurium* colonization [12].

Although our results appear to be in agreement with previous research, they raise new questions regarding the potential mechanism(s) of protection against pathogens involving *B4galnt2*. Indeed, if the heterozygotes experience the same degree of protection against infection as RIIIS/J homozygotes (c_h_ = 0), it implies that the benefit lies in the vascular expression of *B4galnt2* and not in the absence of gastrointestinal expression. However, group C carries a modified RIIIS/J allele that turns off GI expression without turning the vascular expression on [5]. Consequently the RIIIS/J allele would presumably lack the protection provided by vessel expression. This suggests that the RIIIS/J allele might have another, yet unknown function(s) that leads to protection against pathogens. Although further functional characterization of group C alleles is needed, another consideration given their presence in multiple species of the *Mus* genus (an ancestral *M. m. domesticus* population from Iran and *M. spretus*) is that the group C allele class is either ancestral or experienced compensatory evolutionary changes to remove the deleterious effect of blood vessel expression.

Notably, the potential pathogen-driven selection acting on *B4galnt2* in wild mice appears to be similar to the well-documented malaria-driven selection acting on the beta globin gene (HBB) in humans [26, 27], where one allele confers resistance to a pathogen but carries a cost. In the case of *B4galnt2*, the RIIIS/J allele confers resistance to an unknown pathogen(s), while carrying a fitness cost due to prolonged bleeding; for HBB, the HbS allele confers resistance to malaria (*Plasmodium falciparum*) while producing the costly sickle cell phenotype [26, 27]. Where the systems diverge is in the genetic features of the genes. The HBB is a case of overdominance (heterozygote advantage), as heterozygotes have a higher fitness than either homozygote due to increased protection against malaria with a less severe sickling phenotype. Selection operating at *B4galnt2* may be an example of either dominance and/or underdominance, depending on the model considered: when c_h_ = 0, the resistance allele is dominant, and heterozygotes are as protected against infections as the RIIIS/J homozygotes; when c_h_ > 0 however, the resistance allele is co-dominant and heterozygotes are less protected that the RR individuals, and their resulting fitness is less than that of both homozygotes (due to the double cost of infections and bleeding disorder). Interestingly, in the case of the well-studied major histocompatibility complex (MHC) genes, it is often assumed that heterozygote advantage is the leading selective force maintaining the genetic diversity, as dominance alone cannot [28]. However, most studies that set out to determine the nature of selective forces acting on MHC genes failed to identify overdominance, but rather observed signs of rare-allele advantage (or negative frequency-dependence) and/or fluctuating selection, which is modeled here via the exogenous switching of pathogen presence. This indicates that our model is not only valid for the special case of *B4galnt2*, but also for other pathogen-interacting genes.

## Conclusion

In conclusion, by comparing the results of our models to the patterns of polymorphism at *B4galnt2* in natural populations and considering the still limited functional information available for this gene, we are able to recognize the long-term maintenance of the RIIIS/J allele through host-pathogens interactions as a viable hypothesis if its fitness costs due to prolonged bleeding time are roughly half those of being susceptible to a given pathogen. Further, our models identify that a putative dominant-, yet unknown protective function of the RIIIS/J allele appears to be more likely than a protective loss of GI expression in RIIIS/J homozygotes, which may help guide future experiments. Lastly, our model developed here may be used for numerous other biological scenarios, as it does not depend on explicit assumptions regarding a given gene or phenotype, but could be applied to any other diploid model where two co-dominant alleles are maintained by fluctuating selection.

## Methods

### Wild mice

The *Mus musculus domesticus* DNA samples used in this study were derived from previous trapping campaigns [18]. *Mus musculus domesticus* is not a protected species. Permits for catching them were not required at the time they were caught. Individuals were caught on the properties of private landowners, with their oral permission to enter the property and catch mice. Mice were trapped in live traps by experienced personnel. Water-rich food was added in the trap. Straw was placed on the traps for mice to use as nest material, thus providing them with an adequate temperature, and reducing their stress-level. After trapping, mice were sacrificed by CO_2_ inhalation directly in the trap to avoid their handling by the experimenter, thus reducing the human-caused stress to its practical minimum. All procedures were conducted in accordance with German animal welfare law (Tierschutzgesetz) and FELASA guidelines.

We genotyped the *B4galnt2* locus by sequencing a previously developed diagnostic PCR product following the procedure described in Johnsen et al. [4]. Sequences were edited in Seqman (included in DNASTAR, Inc., Madison, Wisc.) and aligned to the homologous sequences from RIIIS/J (GenBank EF372924) and C57BL/6 J (NCBI build 36) using the ClustalW algorithm [29] included in MEGA 4.0.2 [30].

We further typed 12 microsatellite loci located around *B4galnt2* cis-regulatory mutation as described previously [4] (Additional file 6)﻿. The alleles were called with GENEIOUS 7.0 (Biomatters Ltd) and the haplotypic phase was reconstructed with PHASE 2.1 [31]. The algorithm was run 5 times with 10,000 iterations, a thinning interval of 100 and a burn-in of 10.000, and the best output was chosen based on the “goodness of fit”. Microsatellite gene diversity estimates were calculated using GenoDive 2.0 [32]. The two microsatellite loci with highly reduced diversity - located at −30 kb and 0 kb from *B4galnt2* start position - were additionally sequenced using the same primer pairs and PCR conditions as for their typing; the sequencing was performed as for the *B4galnt2* Fragment 5, and the sequences were analyzed in GENEIOUS 7.0 (Biomatters Ltd). Finally, the STRUCTURE analysis included was taken from the output of Linnenbrink et al. [18].

### Model (Additional files 7 and 8)

#### Principle

We modeled the interaction between mouse hosts and pathogens as an evolutionary game [33, 34]. Evolutionary game theory uses mathematical models assuming that a genotype with a high fitness (given by the payoff from the interaction) has a high probability to spread within a population [34]. More precisely, we investigated whether the presence of a pathogen can lead to the maintenance of the two murine alleles of *B4galnt2*. In short, *B4galnt2* is a glycosyltransferase expressed either in the gastrointestinal epithelium (C57BL/6 J allele) or in the vascular endothelium (RIIIS/J allele). Although the second allele causes prolonged bleeding times, most likely at a significant cost to wild mice, both alleles have been maintained by balancing selection for over 2.8 My. Our working hypothesis is that this maintenance may be due to a protective effect of the RIIIS/J allele against pathogen(s), where protection could result from the loss of gastro-intestinal expression and/or from the gain of vascular expression.

#### Pathogen

As our goal is to understand whether the presence of a pathogen can lead to the maintenance of the host alleles in wild populations, we modeled the pathogen as being present with prevalence p. If *p* = 1, the pathogen is overwhelmingly present and every susceptible host is infected; if *p* = 0, no host is infected; if 0 < *p* < 1, the burden of infection for the susceptible hosts is proportional to p. This method allows us to focus on the host population and avoid the many assumptions we should make if we were to model a dynamic and co-evolving pathogen population (e.g. generation time relative to host generation time, population size, transmission mode, transmission efficiency…).

Under the hypothesis of balancing selection due to a trade-off between prolonged bleeding time and pathogen resistance/tolerance, we expect a trench warfare dynamic: (i) the frequency of susceptible hosts increases in the absence of the pathogen due to the cost of resistance, (ii) as the number of susceptible hosts increases, the pathogen population grows, favoring the resistant hosts, (iii) as the number of resistant hosts increases, the pathogen population declines, favoring the susceptible hosts, and the cycle continues [16, 17]. To approximate this phenomenon, we let the environment vary between a state where no pathogen is present (*p* = 0) and a state where pathogens are overwhelming (*p* = 1). This environmental switch (S) is based on the host generations so that the environment changes every S host generations. Varying S allows us to investigate different rates of evolution.

Alternatively, we approximated p with the proportion of susceptible hosts present in the population. This may represent a more “natural” model, as we do not externally force the switch from a pathogenic to non-pathogenic environment. This approximation is thereafter referred to as “frequency dependent environment”.

#### Host

Considering the host population and our focus on the gene *B4galnt2*, we have two allelic states: R represents the vascular endothelium expression allele (RIIIS/J) and C represents the gastrointestinal epithelium expression allele (C57BL/6 J). These alleles can be combined into three possible genotypes - RR, RC and CC - and determine the payoff of an individual. RR individuals carry a cost of bleeding c_b_. CC individuals carry a cost of infection c_i_ in a pathogenic environment and no cost in a pathogen-free environment. RC individuals present an interesting case as they express *B4galnt2* in both tissues, potentially carrying both costs. We previously demonstrated that heterozygous mice display the same bleeding phenotype as the homozygous RR individuals [1]; hence, they carry the same cost of bleeding c_b_. However, we have no evidence that the intestinal phenotype of the heterozygotes is equivalent to that of CC individuals, so we defined a separate infection cost for the heterozygotes c_h_, which is equivalent to the dominance coefficient, h, of the pathogen resistance phenotype, and was explored for three values corresponding to different phenotypes. The first is c_h_ = 0, whereby heterozygotes, like the RR individuals, are not infected by the pathogen. This corresponds to the hypothesis of protection through the gain of vascular expression, i.e. the resistance conferred by the R allele is dominant. The second is c_h_ = c_i_, where heterozygotes are infected to the same degree as the CC individuals. This corresponds to the hypothesis of protection through the loss of gastrointestinal expression, i.e. the resistance allele carried by the R allele is recessive. Finally c_h_ = c_i_/2 represents a state where heterozygotes carry an intermediate resistance phenotype to that of both homozygotes, i.e. the resistance conferred by the R allele is co-dominant.

Following these definitions, we can build the following payoff matrix, where the maximum payoff is 1, and to which the costs of the respective genotypes are withdrawn (similarly to the payoff used by Tellier et al. [35]):

**Table:** 

π	R	C
R	(1-c_b_)	(1-c_b_)*(1-c_h_*p)
C	(1-c_b_)*(1-c_h_*p)	(1-c_i_*p)

In this payoff matrix, c_b_ is the cost of bleeding, c_i_ is the cost of infection for CC individuals, and c_h_ is the cost of infection for heterozygotes. Finally, p is the pathogen prevalence, defined as 0 or 1 in the exogenously changing environment, or the proportion of susceptible hosts in the frequency dependent environment. Finally, we used an exponential fitness mapping, leading to the following fitness matrix:

**Table:** 

*f*	R	C
R	exp(1-c_b_)	exp((1-c_b_)*(1-c_h_*p))
C	exp((1-c_b_)*(1-c_h_*p))	exp(1-c_i_*p)

#### Population dynamics

Our model constrains the host population to a constant size N. We assume that each mouse transmits their strategy at a probability proportional to the fitness of the whole population. This corresponds to an evolutionary game in a Wright-Fisher process [15]. However, since mice are diploid sexual organisms, we added additional steps to the typical haploid “asexual” Wright-Fisher process. First, we selected one individual based on fitness; second we randomly (no mate choice) selected another individual without replacement; third, given the genotypes of the parents, we drew one offspring at random from the set of possible offspring. This process is repeated N times, so that the population always consists of non-overlapping generations. As a result, this method contains 3 N random steps per generation.

Alternatively, we calculated the expected genotypes of the offspring population from the parent population weighted by its fitness, using Hardy-Weinberg equilibrium (HWE). First we calculated the weight W of each genotype according to their population frequencies P and their fitness *f* (1). Then we calculated the weighted allele frequencies A (2), and finally the offspring genotype frequencies O (3).

1 $$ {\displaystyle \begin{array}{l}{\mathrm{W}}_{\mathrm{RR}}={\mathrm{P}}_{\mathrm{RR}}\ast {f}_{\mathrm{RR}}\hfill \\ {}{\mathrm{W}}_{\mathrm{RC}}={\mathrm{P}}_{\mathrm{RC}}\ast {f}_{\mathrm{RC}}\hfill \\ {}{\mathrm{W}}_{\mathrm{CC}}={\mathrm{P}}_{\mathrm{CC}}\ast {f}_{\mathrm{CC}}\hfill \end{array}} $$

2 $$ {\displaystyle \begin{array}{l}{\mathrm{A}}_{\mathrm{C}}=\left({\mathrm{W}}_{\mathrm{C}\mathrm{C}}\ast 2+{\mathrm{W}}_{\mathrm{R}\mathrm{C}}\right)/2\mathrm{N}\hfill \\ {}{\mathrm{A}}_{\mathrm{R}}=\left({\mathrm{W}}_{\mathrm{R}\mathrm{R}}\ast 2+{\mathrm{W}}_{\mathrm{R}\mathrm{C}}\right)/2\mathrm{N}\hfill \end{array}} $$

3 $$ {\displaystyle \begin{array}{l}{\mathrm{O}}_{\mathrm{R}\mathrm{R}}={{\mathrm{A}}_{\mathrm{R}}}^2\ast \mathrm{N}\hfill \\ {}{\mathrm{O}}_{\mathrm{R}\mathrm{C}}=2\ast {\mathrm{A}}_{\mathrm{R}}\ast {\mathrm{A}}_{\mathrm{C}}\ast \mathrm{N}\hfill \\ {}{\mathrm{O}}_{\mathrm{C}\mathrm{C}}={{\mathrm{A}}_{\mathrm{C}}}^2\ast \mathrm{N}\hfill \end{array}} $$

As this calculation creates non-integer values, the results were rounded to the next lower integer and the difference to N was adjusted by randomly adding/removing individuals of any genotype. Hence this method contains only few random steps per generation.

Finally, the new generation - obtained either by the HWE-based or the Wright-Fisher like process - is allowed to mutate with a probability μ taken from a Poisson distribution. In our case, this is a proxy for migration, as the two *B4galnt2* alleles are highly divergent and thus unlikely to easily mutate from one state to the other.

#### Simulations (Additional file 9)

We ran every model with a local population size of 500 and a mutation rate of 0.005 over 10,000 generations. All simulations were started with a random population that consisted of roughly 1/3 of each genotype. We varied the genotype-specific costs c_b_, c_h_ and c_i_, the environmental switch S, the starting environment and the definition of the pathogen. Each parameter combination was repeated 50 to 100 times. All the parameter combinations tested are summarized below:

**Population Dynamics: Wright-Fisher process with random mating**

- c_h_ = 0, c_h_ = c_i_/2, c_h_ = c_i_
- c_b_ and c_i_ from 0 to 1 by 0.2 steps
- Mutation rate: μ = 0.005
- Environment:
  - o Constant: 100 iterations for both environments
  - o Frequency-dependent: 100 iterations
  - o Switching environment:
    - Switch frequencies: 1, 10, 50, 100, 500, 1000, 5000
    - 50 iterations starting with a pathogenic environment +50 starting with a non-pathogenic environment

**Population Dynamics: HWE-based process**

- c_h_ = 0, c_h_ = c_i_/2, c_h_ = c_i_
- c_b_ and c_i_ from 0 to 1 by 0.05 steps
- Mutation rate: μ = 0, μ = 0.005
- Environment:
  - o Constant: 100 iterations for both environments
  - o Frequency-dependent: 100 iterations
  - o Switching environment:
    - Switch frequencies: 1, 10, 50, 100, 250, 500, 750, 1000
    - 100 iterations starting with a pathogenic environment +100 starting with a non-pathogenic environment

#### Results

We present two aspects of the model results: first the average population frequencies are the frequencies of each genotype in the host population averaged across the 10,000 generations and across the 50 to 100 repetitions; second, the comparison to the real data consists of the average similarity (Si) between the simulated (F) and the observed (O) population frequencies for each genotype (RR for RIIIS/J homozygotes, CC for C57BL/6 J homozygotes and RC for heterozygotes):

$$ \mathrm{Si}=1\hbox{-} \left(\mathrm{abs}\left({\mathrm{F}}_{\mathrm{RR}}\hbox{-} {\mathrm{O}}_{\mathrm{RR}}\right)+\mathrm{abs}\left({\mathrm{F}}_{\mathrm{RC}}\hbox{-} {\mathrm{O}}_{\mathrm{RC}}\right)+\mathrm{abs}\left({\mathrm{F}}_{\mathrm{CC}}\hbox{-} {\mathrm{O}}_{\mathrm{CC}}\right)\right)/3 $$

The figures were produced in R using the reshape [36] and corrplot [37] packages.

## Additional files

Additional file 1: Figure S1. Average genotype frequencies in the model with a constant environment. The frequencies are displayed according to the cost of bleeding (y axis) and of infection (x axis). For the non-pathogenic environment (left panel), all ch values are equivalent since ci = ch = 0 in all cases. For the pathogenic environment however, the different models of ch values are shown. The average genotype frequencies across 100 simulations using the HWE; process, each with 10,000 generations, are displayed. The frequencies are color-coded according to the legend on the right. Stars indicate an excess of homozygotes. (PDF 192 kb)

Additional file 2: Figure S2. Average genotype frequencies in the model with a switching environment. The frequencies are displayed according to the frequency of environmental change expressed in host generations, the cost of bleeding (y axis) and of infection (x axis). The average genotype frequencies across 200 simulations using the HWE-process, each with 10,000 generations, are displayed for ch = 0 (A), ch = ci/2 (B) and ch = ci (C). The frequencies are color-coded according to the legend on the right. Stars indicate an excess of homozygotes. (PDF 469 kb)

Additional file 3: Figure S4. Average genotype frequencies in the model with a frequency-dependent environment. The frequencies are displayed according to the value of ch, the cost of bleeding (y axis) and of infection (x axis). The average genotype frequencies across 100 simulations using the HWE-process, each with 10,000 generations, are displayed. The frequencies are color-coded according to the legend on the right. Stars indicate an excess of homozygotes. (PDF 152 kb)

Additional file 4: Figure S3. Similarity of the populations simulated with the HWE-process to the natural populations. A) Similarity to populations from Group A, B) Similarity to populations from Group B, C) Similarity to populations from Group C. The similarity is displayed according to the value of ch, the cost of bleeding (y axis) and of infection (x axis), and the modeled environment (constant with or without pathogen, and switching between pathogenic and non pathogenic every 1, 50 or 500 host generations). The similarity is color-coded according to the legend on the right. Stars indicate an excess of homozygotes. Full similarity is achieved when all genotype frequencies coincide. (PDF 1079 kb)

Additional file 5: Figure S5. Similarity of the natural populations to the populations simulated in the model with a frequency-dependent environment, using the HWE-process. The similarity is displayed according to the value of ch, the cost of bleeding (y axis) and of infection (x axis). The similarity is color-coded according to the legend on the right. Stars indicate an excess of homozygotes. (PDF 197 kb)

Additional file 6: Table S1. Microsatellite data. Repeat number for the 12 microsatellite markers linked to *B4galnt2*. (XLSX 62 kb)

Additional file 7: Model. Python code of the model used in this study. (PY 47 kb)

Additional file 8: Model ReadMe. Description of the model parameters and output formats to ease the use of the python code. (PDF 92 kb)

Additional file 9: Simulated Average Populations. Text file containing the results of all simulations done in this study to allow for direct comparison with natural datasets without the need to rerun the program. (ZIP 10672 kb)

## Abbreviations

CC individuals: Individuals homozygote for C57BL/6 J allele
HWE: Hardy-Weinberg Equilibrium
RC individuals: Individuals heterozygotes for the RIIIS/J and C57BL/6 J allele
RR individuals: Individuals homozygote for the RIIIS/J allele
VWD: Von Willebrand disease
VWF: Von Willebrand factor
