# Supplementary material for: Evaluating the maintenance of disease-associated variation at the blood group-related gene B4galnt2 in house mice
Source: BMC Evol Biol. 2017 Aug 14;17:187. doi: 10.1186/s12862-017-1035-7 (PMC5557512; doi:10.1186/s12862-017-1035-7)
Supplement: Supplementary file 1 — Average genotype frequencies in the model with a constant environment. The frequencies are displayed according to the cost of bleeding (y axis) and of infection (x axis). For the non-pathogenic environment (left panel), all ch values are equivalent since ci = ch = 0 in all cases. For the pathogenic environment however, the different models of ch values are shown. The average genotype frequencies across 100 simulations using the HWE; process, each with 10,000 generations, are displayed. The frequencies are color-coded according to the legend on the right. Stars indicate an excess of homozygotes. (PDF 192 kb) [file 12862_2017_1035_MOESM1_ESM.pdf]

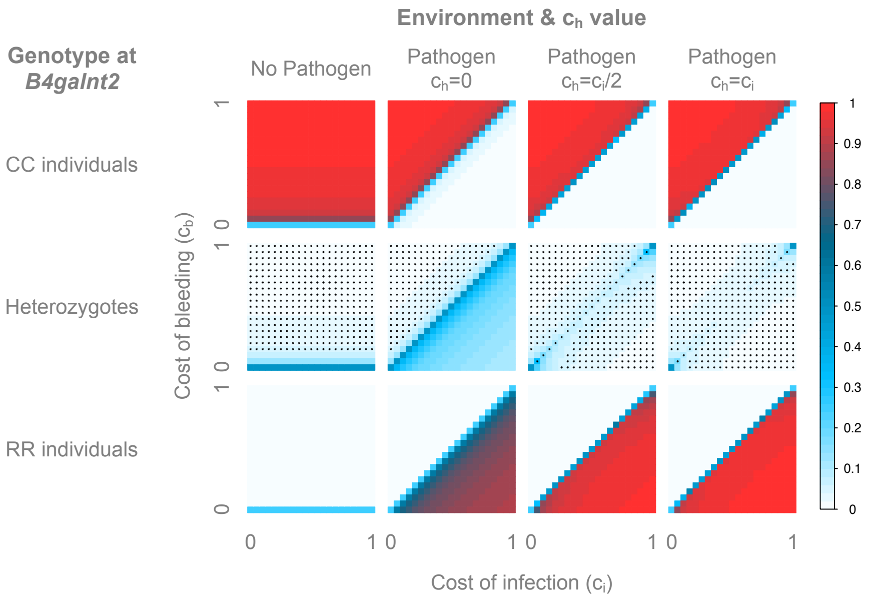

Figure S1: Average genotype frequencies in the model with a constant environment. The frequencies are displayed according to the cost of bleeding (y axis) and of infection (x axis). For the non-pathogenic environment (left panel), all c_h values are equivalent since c_i=c_h=0 in all cases. For the pathogenic environment however, the different models of c_h values are shown. The average genotype frequencies across 100 simulations using the HWE-process, each with 10000 generations, are displayed. The frequencies are color-coded according to the legend on the right. Stars indicate an excess of homozygotes.
