# Supplementary material for: Evaluating the maintenance of disease-associated variation at the blood group-related gene B4galnt2 in house mice
Source: BMC Evol Biol. 2017 Aug 14;17:187. doi: 10.1186/s12862-017-1035-7 (PMC5557512; doi:10.1186/s12862-017-1035-7)
Supplement: Supplementary file 2 — Average genotype frequencies in the model with a switching environment. The frequencies are displayed according to the frequency of environmental change expressed in host generations, the cost of bleeding (y axis) and of infection (x axis). The average genotype frequencies across 200 simulations using the HWE-process, each with 10,000 generations, are displayed for ch = 0 (A), ch = ci/2 (B) and ch = ci (C). The frequencies are color-coded according to the legend on the right. Stars indicate an excess of homozygotes. (PDF 469 kb) [file 12862_2017_1035_MOESM2_ESM.pdf]

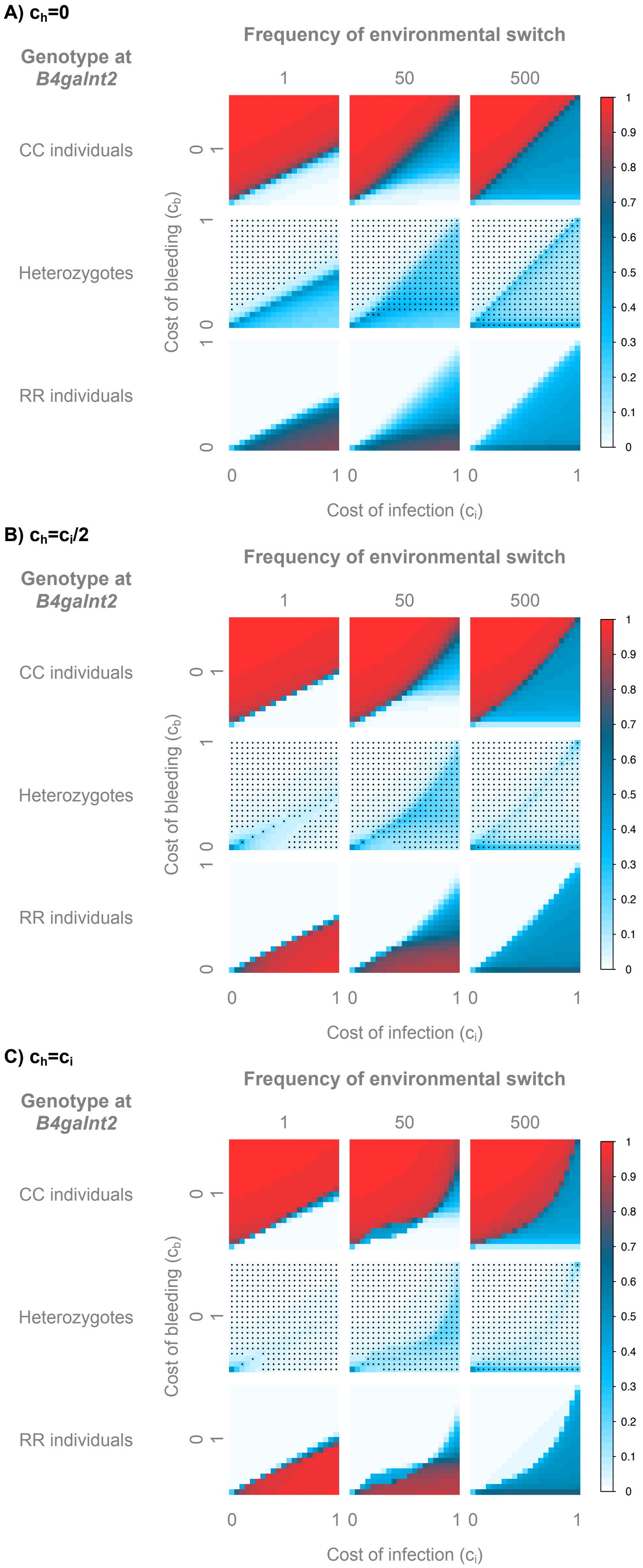


**Figure S2: Average genotype frequencies in the model with a switching environment.** The frequencies are displayed according to the frequency of environmental change expressed in host generations, the cost of bleeding (y axis) and of infection (x axis). The average genotype frequencies across 200 simulations using the HWE-process, each with 10000 generations, are displayed for $c_h=0$ (A), $c_h=c_i/2$ (B) and $c_h=c_i$ (C). The frequencies are color-coded according to the legend on the right. Stars indicate an excess of homozygotes.
