# Supplementary material for: Evaluating the maintenance of disease-associated variation at the blood group-related gene B4galnt2 in house mice
Source: BMC Evol Biol. 2017 Aug 14;17:187. doi: 10.1186/s12862-017-1035-7 (PMC5557512; doi:10.1186/s12862-017-1035-7)
Supplement: Supplementary file 3 — Average genotype frequencies in the model with a frequency-dependent environment. The frequencies are displayed according to the value of ch, the cost of bleeding (y axis) and of infection (x axis). The average genotype frequencies across 100 simulations using the HWE-process, each with 10,000 generations, are displayed. The frequencies are color-coded according to the legend on the right. Stars indicate an excess of homozygotes. (PDF 152 kb) [file 12862_2017_1035_MOESM3_ESM.pdf]

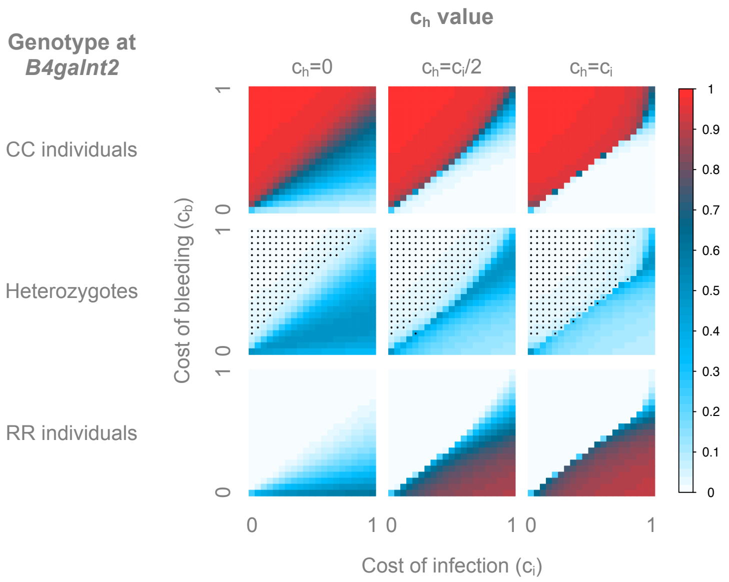

Figure S4: Average genotype frequencies in the model with a frequency-dependent environment. The frequencies are displayed according to the value of c_h, the cost of bleeding (y axis) and of infection (x axis). The average genotype frequencies across 100 simulations using the HWE-process, each with 10000 generations, are displayed. The frequencies are color-coded according to the legend on the right. Stars indicate an excess of homozygotes.
