# Supplementary material for: Evaluating the maintenance of disease-associated variation at the blood group-related gene B4galnt2 in house mice
Source: BMC Evol Biol. 2017 Aug 14;17:187. doi: 10.1186/s12862-017-1035-7 (PMC5557512; doi:10.1186/s12862-017-1035-7)
Supplement: Supplementary file 4 — Similarity of the populations simulated with the HWE-process to the natural populations. A) Similarity to populations from Group A, B) Similarity to populations from Group B, C) Similarity to populations from Group C. The similarity is displayed according to the value of ch, the cost of bleeding (y axis) and of infection (x axis), and the modeled environment (constant with or without pathogen, and switching between pathogenic and non pathogenic every 1, 50 or 500 host generations). The similarity is color-coded according to the legend on the right. Stars indicate an excess of homozygotes. Full similarity is achieved when all genotype frequencies coincide. (PDF 1079 kb) [file 12862_2017_1035_MOESM4_ESM.pdf]

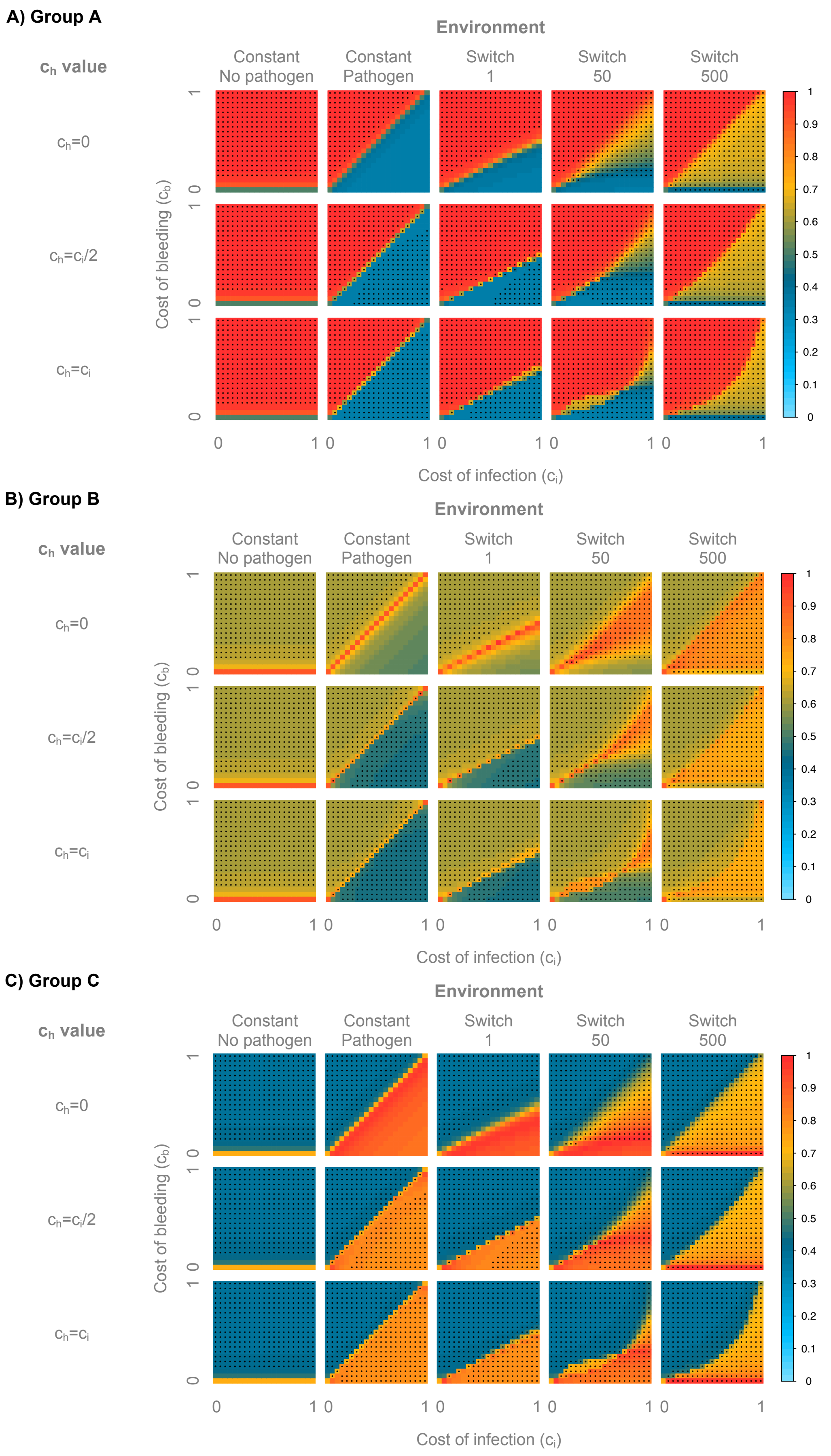

Figure S3: Similarity of the populations simulated with the HWE-process to the natural populations. A) Similarity to populations from Group A, B) Similarity to populations from Group B, C) Similarity to populations from Group C. The similarity is displayed according to the value of c_h, the cost of bleeding (y axis) and of infection (x axis), and the modeled environment (constant with or without pathogen, and switching between pathogenic and non pathogenic every 1, 50 or 500 host generations). The similarity is color-coded according to the legend on the right. Stars indicate an excess of homozygotes. Full similarity is achieved when all genotype frequencies coincide.
