# Supplementary material for: Evaluating the maintenance of disease-associated variation at the blood group-related gene B4galnt2 in house mice
Source: BMC Evol Biol. 2017 Aug 14;17:187. doi: 10.1186/s12862-017-1035-7 (PMC5557512; doi:10.1186/s12862-017-1035-7)
Supplement: Supplementary file 5 — Similarity of the natural populations to the populations simulated in the model with a frequency-dependent environment, using the HWE-process. The similarity is displayed according to the value of ch, the cost of bleeding (y axis) and of infection (x axis). The similarity is color-coded according to the legend on the right. Stars indicate an excess of homozygotes. (PDF 197 kb) [file 12862_2017_1035_MOESM5_ESM.pdf]

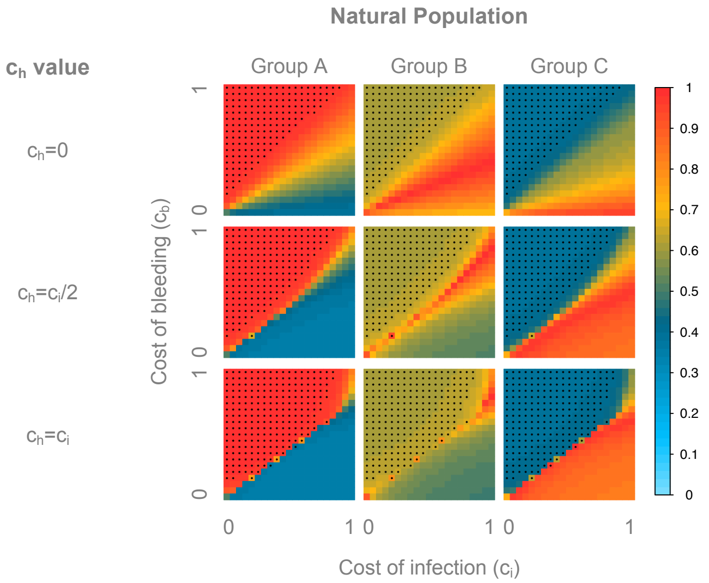

Figure S5: Similarity of the natural populations to the populations simulated in the model with a frequency-dependent environment, using the HWE-process. The similarity is displayed according to the value of c_h, the cost of bleeding (y axis) and of infection (x axis). The similarity is color-coded according to the legend on the right. Stars indicate an excess of homozygotes.
