# Supplementary material for: Evaluating the maintenance of disease-associated variation at the blood group-related gene B4galnt2 in house mice
Source: BMC Evol Biol. 2017 Aug 14;17:187. doi: 10.1186/s12862-017-1035-7 (PMC5557512; doi:10.1186/s12862-017-1035-7)
Supplement: Supplementary file 8 — Model ReadMe. Description of the model parameters and output formats to ease the use of the python code. (PDF 92 kb) [file 12862_2017_1035_MOESM8_ESM.pdf]

Additional File 2: Model ReadMe

Citation

When using, please cite:
Exploring the persistence of von Willebrand like bleeding disorder in wild populations of Mus sp. Vallier et al. 2017, BMC Evolutionary Biology.

Requirements

The model is written in **python 2.7** and requires the following modules:
numpy, random, math, decimal, sys, datetime.
Additionally, when using **output** option 7, matplotlib and pylab are also needed.

Options

All options **must be specified**; "-1" can be used for irrelevant options.

Fitness:

cb: cost for the R3 allele - *float between 0 and 1*
Applied to R3 homozygotes and heterozygotes

ci2: cost for the B6 allele - *float between 0 and 1*
Applied only to B6 homozygotes

pathogen: sets the heterozygotes' cost **ci1** for the B6 allele - *character "gut", "dosage" or "blood"*
"gut" ci1=ci2
"dosage" ci1=ci2/2
"blood" ci1=0

mat_model: fitness matrix model - *character: "linear", "exp" or "div"*
"linear" fitness=1-cost
"exp" fitness=exp(1-cost)
"div" fitness=1/(cost+b) # b is a baseline cost defined by the **shift** option

shift: the baseline cost when **mat_model** is "div" - *any non-zero number*

Population:

fR: initial R3 allele frequency - *float between 0 and 1 for defined starting frequency, -1 for random start*

N: population size - *positive integer*

u: mutation rate - *float between 0 and 1*

nextgen: population dynamics model - *character: "HW", "random" or "randomsex"*
"HW" uses the Hardy-Weinberg equilibrium to calculate each generation based on the previous one weighted by its fitness
"random" Wright-Fisher process *!computation time directly correlated to population size N!*
"randomsex" modified Wright-Fisher process: picks two individuals from the current generation, one based on fitness, one randomly and select one offspring at random among the possible offspring given the genotype of the parents *!computation time directly correlated to population size N!*

Environment:

ENV: initial environment - *integer either 0 or 1*
0 ➔ no pathogen: ci2=ci1=0 regardless of their defined values
1 ➔ pathogen: ci2 and ci1 as defined in the corresponding options

switchrate: switch rate for changing the environment between 0 and 1 - *positive integer (0 is a constant environment)*

switch_option: how to switch the environment - *character: "constant", "average" or "max"*
"constant" will switch the environment exactly every **switch_option** generations
"average" will switch on average every **switch_option** generations
"max" will switch maximally every **switch_option** generations

cost: how to integrate the environment effect on the fitness costs ci2 and ci1 - *character: "constant" or "frequency"*
"constant" the fitness is constant and only depends on the environment as described for **ENV**
"frequency" the fitness is dependent on the frequency of pathogen (p): ci2=*ci2**p ci1=*ci1**p, with p defined as the proportion of susceptible hosts in the population

Run parameters:

run: length of the run (=generations) - *positive integer*

repeat: number of iterations - *positive integer*

output: output option - *character, one or a combination of integers between 1 and 7 (e.g output="1", output="15", output="271")*
"1" export only the average genotype frequencies, makes one file per parameter combination with all repeats inside
"2" export all genotype frequencies along the **run** generations, produce one file per iteration
"3" export the last time heterozygotes were observed in the population, makes one file per parameter combination with all repeats inside
"4" export the last time R3 homozygotes were observed in the population, makes one file per parameter combination with all repeats inside
"5" export the # of generations with genotype frequency >90%, makes one file per parameter combination with all repeats inside
"6" export the # of generations with genotype frequency >60%, makes one file per parameter combination with all repeats inside
"7" plot genotype frequencies along the **run** generations, produce one file per iteration *!only works together with **output**=2!*

path: path to the output folder - *character "computer" or the complete path to the output folder*

arrayID: to set the random number generator - positive integer

Run

The model can be run from the terminal by giving the option values in the following precise order:

python B4GALNT2_model.py $cb $ci2 $fR $N $nextgen $u $ENV $switchrate $switch_option $run $output $path $repeat $mat_model $shift $pathogen $cost $arrayID

Alternatively, the use of for loops in shell scripts is possible to run multiple parameter values.

Output format

Output #1:

Name: average_population_cb_ci1_ci2_fR_N_nextgen_u_ENV_switchrate_ *switch_option*_run_*mat_model*_shift_pathogen_cost.txt
Contains 5 (**cost**=”constant”) or 6 (**cost**=”frequency”) columns: cb, ci2, average R3 homozygote frequency, average heterozygote frequency, average B6 homozygote frequency, average pathogen frequency (if **cost**=”frequency”)

Output #2:

Name: population_change_cb_ci1_ci2_fR_N_nextgen_u_ENV_switchrate_ *switch_option*_run_*mat_model*_shift_pathogen_cost_repeat.txt
Contains 4 (cost=”constant”) or 5 (cost=”frequency”) columns: Generation, R3 homozygote frequency, heterozygote frequency, B6 homozygote frequency, pathogen frequency (if **cost**=”frequency”)

Output #3:

Name: last_Het_cb_ci1_ci2_fR_N_nextgen_u_ENV_switchrate_ *switch_option*_run_*mat_model*_shift_pathogen_cost.txt
Contains 6 columns: cb, ci2, generation, R3 homozygote frequency, heterozygote frequency, B6 homozygote frequency

Output #4:

Name: last_R3_cb_ci1_ci2_fR_N_nextgen_u_ENV_switchrate_ *switch_option*_run_*mat_model*_shift_pathogen_cost.txt
Contains 6 columns: cb, ci2, generation, R3 homozygote frequency, heterozygote frequency, B6 homozygote frequency

Output #5:

Name: fixation_cb_ci1_ci2_fR_N_nextgen_u_ENV_switchrate_ *switch_option*_run_*mat_model*_shift_pathogen_cost.txt
Contains 5 columns: cb, ci2, #generations that R3 homozygotes were >90% of the population, #generations that heterozygotes were >90% of the population, #generations that B6 homozygotes were >90% of the population

Output #6:

Name: relaxed_fixation_cb_ci1_ci2_fR_N_nextgen_u_ENV_switchrate_ *switch_option*_run_*mat_model*_shift_pathogen_cost.txt
Contains 5 columns: cb, ci2, #generations that R3 homozygotes were >60% of the population, #generations that heterozygotes were >60% of the population, #generations that B6 homozygotes were >60% of the population

Output #7:

Name: population_change_cb_ci1_ci2_fR_N_nextgen_u_ENV_switchrate_ *switch_option*_run_*mat_model*_shift_pathogen_cost_repeat.pdf
